# The causal role of gastroesophageal reflux disease in endometriosis: a bidirectional Mendelian randomization study

**DOI:** 10.3389/fmed.2024.1440157

**Published:** 2024-10-30

**Authors:** Zunlin Shi, Zhi Li, Kana Wang, Fan Yang

**Affiliations:** ^1^College of Electronics and Information Engineering, University of Sichuan, Chengdu, China; ^2^Department of Gynecology and Obstetrics, West China Second Hospital, University of Sichuan, Chengdu, China; ^3^Key Laboratory of Obstetric and Gynecologic and Pediatric Diseases and Birth Defects of Ministry of Education, West China Second Hospital, University of Sichuan, Chengdu, China

**Keywords:** bidirectional Mendelian randomization (MR) analysis, gastroesophageal reflux disease, risk of developing endometriosis, endometriosis confined to the uterus, endometriosis

## Abstract

Observational studies have reported an association between gastroesophageal reflux disease (GERD) and endometriosis. We conducted a two-sample and bidirectional Mendelian randomization analysis to determine whether those associations are causal. Two-sample and bidirectional MR analyses were performed using summary statistics from the European Individual Genome-Wide Association Study (GWAS). The inverse variance weighting (IVW) method is used as the main analysis method to evaluate causality. Sensitivity analyses were performed to assess heterogeneity, horizontal versatility, and stability. The results showed no significant causal association between GERD in women with endometriosis in the UK Bank database [ratio (OR) ≈ 0, 95% adjusted interval (CI) 1.0007∼1.0044, *P* = 0.006] and Finn databases [ratio (OR) = 1.29, 95% adjusted interval (CI) 0.99∼1.67, *P* = 0.06]. However, when studying the Finn database only for endometriosis, which is confined to the uterus, a significant increase in GERD was limited to the risk of endometriosis in the uterus [ratio (OR) = 1.47, 95% adjusted interval (CI) 1.00∼2.17, *P* = 0.05]. Sensitivity analysis showed that the results were robust and did not detect multi efficacy or heterogeneity. Meanwhile, reverse MR analysis showed that endometriosis did not increase the risk of GERD. This MR study supports a causal relationship between GERD and an increased risk of endometriosis confined to the uterus. Therefore, patients with gastric esophageal reflux should be treated with gynecological examination to avoid and prevent the development of endometriosis.

## 1 Introduction

Endometriosis is a common benign disease in gynecology, affecting approximately 10% (190 million) of women and girls of childbearing age worldwide (1). It is a chronic disease that is affected by estrogen regulation and is associated with dysmenorrhea, sexual intercourse, bowel pain and/or urination pain, chronic pelvic pain, bloating, nausea, and fatigue, and some patients also suffer from depression, anxiety and infertility (2). Patients bear a severe burden of life and psychology, with enormous social and economic burdens (3–6). In addition, endometriosis sufferers often experience symptoms of intestinal or bladder irritation due to chronic pain comorbidities, which overlap with other diseases, leading to significant delays in the diagnosis of endometriosis after the onset of symptoms (7). Therefore, it is important to explore the factors associated with endometriosis to guide the early diagnosis and treatment of the disease.

Gastroesophageal reflux disease (GERD) refers to the reflux of gastroduodenal contents into the esophagus causing acid reflux, heartburn and other symptoms. Reflux can cause tissue damage to the mouth, throat, and bronchial tract and other tissue damage near the esophagus. Esophageal manifestations include asthma, chronic cough, idiopathic pulmonary fibrosis, hoarseness, chronic sore throat and tooth erosion (8). These nonspecific symptoms can cause overlap or confusion with other diseases (8). A clinical report in the United States showed that after long-term gastric esophageal reflux treatment, patients with GERD had a history of endometriosis and endometriosis resection and showed continued progression of symptoms of dysphagia, vomiting and reflux, and weight loss, with unknown causes and complications (9). The American Gastroenterological Society study also suggests that intestinal endometriosis can present with acute abdominal pain and small intestinal obstruction on CT. Therefore, when women of childbearing age have acute abdominal pain, the possibility of endometriosis involving the gastrointestinal tract should be considered (10, 11). Over the past five years, the American Gastrointestinal Association has also reported a possible association between a history of GERD and a history of hysterectomy in women (12). In addition, in recent new drug reports, domestic and foreign research institutes and companies have reported the invention of novel prevention and treatment drugs for both endometriosis and gastrointestinal diseases, which have synergistic effects (13–15). Although the underlying mechanisms of these phenomena are unclear, some evidence may support the potential of endometriosis to cause GERD, which in turn can lead to elevated levels of inflammation, leading to the development of endometriosis.

Although clinical observations and some current evidence suggest a possible association between GERD and endometriosis, it has not been possible to establish a causal link between them. Mendelian randomization (MR) is an innovative approach to optimizing observational epidemiology and can be used to investigate the causal effects of altered exposure on health outcomes (16).

The method introduces instrumental variables that affect exposure only, independent of potential confounding factors associated with outcomes and exposure outcomes, and will use single nucleotide polymorphisms (SNPs), which are highly correlated with exposure and randomized genetic variation, as instrumental variables to assess the causal relationship between the variable exposure and the outcome (17). SNPs have characteristics that precede disease occurrence and are unaffected by the outcome and the correlation between many confusing exposures and outcomes; thus, MR studies can reduce the risk of potential bias from confounding factors and reverse causation and effectively evaluate the causal relationship between exposure and outcome (18). To date, only one study of the correlation between GERD and endometriosis using MR has been retrieved (19). However, the study did not address the correlation between endometriosis and GERD at different sites. Therefore, this study explores the causal relationship between GERD and endometriosis through MR and further explores the correlation between endometriosis at different sites. It seeks a new research direction for exploring the pathogenesis of endometriosis at different sites and provides a theoretical basis for endometriosis screening and early accurate diagnosis of endometriosis in patients with GERD.

## 2 Materials and methods

### 2.1 GWAS summary-level data of GERD and endometriosis

**FIGURE 1:**
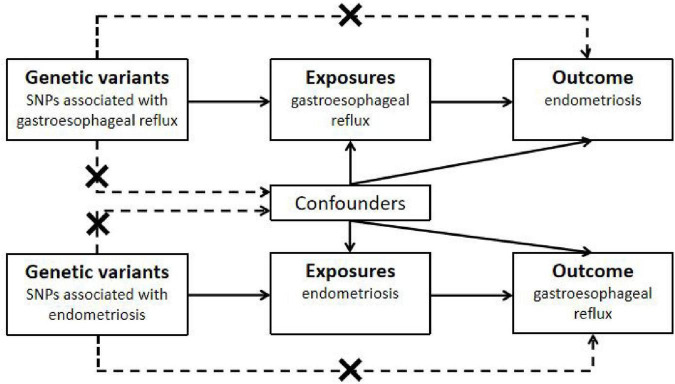
The overall flow chart of the bidirectional MR study. SNPs, single nucleotide polymorphisms. There are three assumptions of Mendelian randomization design. The first assumption is that the genetic variants used as instrumental variables should be robustly associated with the exposure; the second assumption is that the used genetic variants should not be associated with any confounders; and the third assumption is that the selected genetic variants should affect the risk of the outcome merely through the risk factor, not via alternative pathways.

**FIGURE 2:**
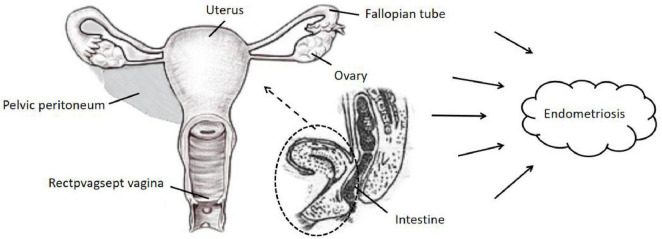
Classification of endometriosis occurring at different sites. The main possible locations where endometriosis occurs in the tissues surrounding the body of the uterus in women: pelvic peritoneum, rectpvagsept vagina, fallopian tubes, uterus, ovaries, intestine, etc.

**FIGURE 3:**
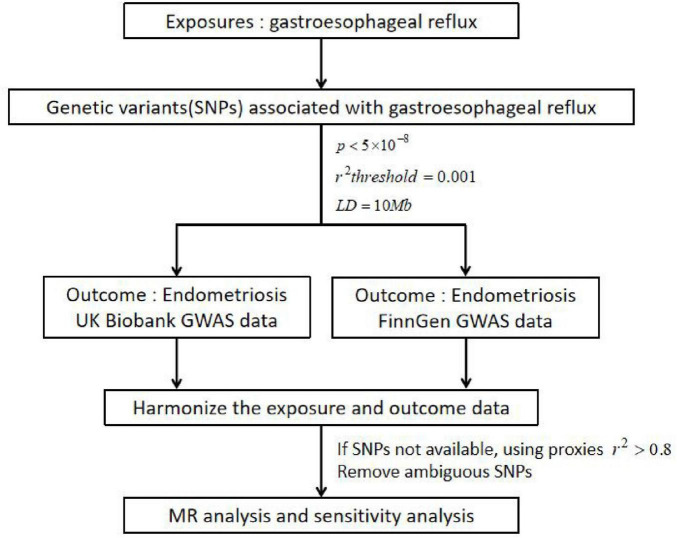
Specific analysis process. Example: GERD as a exposure/endometriosis as an outcome example; GERD, gastroesophageal reflux disease; SNPs, single nucleotide polymorphisms; *p*, statistical *p*-value; *r*^2^, correlation index for evaluating the effect of the fitted regression; *LD*, linkage disequilibrium; GWAS, genome-wide association study. GERD was selected as the exposure and endometriosis was selected as the outcome. The screened instrumental variable (SNPs) was associated with exposure, fulfilling the following three conditions: *p* < 5×10^−8^, *r*^2^*threshold* = 0.001 and *LD=10Mb*. In addition, the aggregate GWAS statistics for endometriosis are from FinnGen database (8,288 cases of endometriosis and 9,972 cases of health control) and UK Bank database (1,496 cases of endometriosis). If SNPs not available, using proxies *r*^2^ > 0.8, remove ambiguous SNPs and harmonize the exposure and outcome data. Finally, MR analysis and sensitivity analysis were performed.

The overall flow chart of the bidirectional MR study is shown in Figure 1. A genome-wide association study (GWAS) meta-analysis was used to study European GERD data, which included 78,707 patients with GERD in Europe and 288,734 healthy controls (20). These data are available in the GWAS Catalog project database (21). In addition, the aggregate GWAS statistics for endometriosis are from the FinnGen database (8,288 cases of endometriosis and 9,972 cases of healthy controls) and the UK Bank database (1,496 cases of endometriosis) (22). Among them, the FinnGen database includes subsets of endometriosis occurring in fallopian tubes (116 cases of endometriosis and 146 cases of healthy controls), the uterus (2,372 cases of endometriosis and 1,600 cases of healthy controls), the pelvic peritoneum (2,953 cases of endometriosis and 3,940 cases of healthy controls), the ovaries (3,231 cases of endometriosis and 3,865 cases of healthy controls), the rectal vaginal and vaginal compartments (1,360 cases of endometriosis and 1,570 cases of healthy controls) and the intestines (117 cases of endometriosis and 375 cases of healthy controls). Table 1 provides details of the GWAS summary level data of exposure and outcome analyzed in this MR study. All data analyzed in this study were obtained from publicly available databases in which ethical approval was obtained for each cohort, and informed consent was obtained from all participants prior to participation. Figure 2 shows endometriosis at different sites. The specific analysis process (Example: GERD as an exposure/endometriosis as an outcome example) is shown in Figure 3.

**TABLE 1:** Details of the GWAS summary-level data.

Traits	*N* case	*N* control	Population	Data accession address
GERD	129,080	473,524	European	https://gwas.mrcieu.ac.uk/
ENDOMETRIOSIS (UK Bank database)	1,496	446,991	European	http://www.nealelab.is/uk-biobank
ENDOMETRIOSIS (FinnGen database)	8,288	68,969	European	https://r9.finngen.fi/
ENDOMETRIOSIS_FALLOPIAN_TUBE	116	68,969	European	https://r9.finngen.fi/
ENDOMETRIOSIS_UTERUS	2,372	68,969	European	https://r9.finngen.fi/
ENDOMETRIOSIS_PELVICPERITONEUM	2,953	68,969	European	https://r9.finngen.fi/
ENDOMETRIOSIS_OVARY	3,231	68,969	European	https://r9.finngen.fi/
ENDOMETRIOSIS_RECTPVAGSEPT_VAGINA	1,360	68,969	European	https://r9.finngen.fi/
ENDOMETRIOSIS_INTESTINE	117	68,969	European	https://r9.finngen.fi/

GERD, gastroesophageal reflux disease.

### 2.2 Selection of instrumental variables

Mendelian randomization is a method of studying the causal relationship between exposure and outcome using genetic variation as an instrumental variable in medical research observations (23). Instrumental variable (IV) selection satisfies correlation with exposure, and IVs should be independent of any confusion associated with the exposure result, which means that there are no causal pathways from IVs to results, except through exposure (24, 25).

The selection of gene variants involves controlling genome-wide significance thresholds (*p* < 5×10^−8^) and screening SNPs as IVs for MR analysis (16). Consideration of chained unbalanced SNPs had an impact on the resulting effect values by removing SNPs with *r*^2^ < 0.001 to the most significant SNP in the 10,000*kb* range of chromosomes to satisfy near-perfect chained equilibrium between the two SNPs and to ensure the independence of each instrumental variable. Additionally, palindromic SNPs, outcome-associated SNPs (*p* < 0.05), and SNPs not present in the resultant GWAS pooled data were removed. The extent of weak instrumental bias was assessed according to the f-statistic formula, and IVs with *F* > 10 were retained to avoid bias caused by weak IVs (26).

Body mass index, height, depression and anxiety, menarche, reproductive history, back pain, and the influence of economic factors may be potential confounders affecting GERD and endometriosis (22, 27–34). To increase the credibility of the findings, SNPs associated with these confounders (*p* < 5×10^−8^) were retrieved from the IEU Open GWAS program database and excluded, and the number of these confounding accessions is shown in Table 2.

**TABLE 2:** Sources of confounding factors.

Confounding factors	Sources
Height	https://doi.org/10.1093/humrep/dex207 (27)
Body mass index	https://doi.org/10.1016/j.ejogrb.2004.11.019 (28)
Depression and anxiety	https://doi.org/10.2147/vhrm.s147173 (29)
Depression and anxiety	https://doi.org/10.1001/jamanetworkopen.2022.51214 (22)
Age at menarche	https://doi.org/10.1007/s00404-022-06541-0 (30)
Age at menarche	https://doi.org/10.1016/j.fertnstert.2012.05.035 (31)
Reproductive history	https://doi.org/10.1097/01.AOG.0000142714.54857.f8 (32)
Back pain	https://doi.org/10.3389/fncel.2020.590823 (33)
The influence of economic factors	https://doi.org/10.1093/oxfordjournals.epirev.a036270 (34)

### 2.3 Statistical methods

The MR study relied on three core instrumental variable assumptions (correlation with exposure, independence from confounders, and exclusion of restrictions unrelated to outcome) to test the causal effect of exposure on outcome (16). Inverse variance weighted (IVW) analysis was used to estimate the causal effect of exposure and outcome using the Wald ratio estimator based on the principles of meta-analysis (35). To demonstrate the stability and directionality of the results, in addition to the IVW method, two other MR methods [MR-Egger method and weighted median method] were used to assess causality. The MR-Egger method estimates the causal effect of genes on traits by fitting a linear regression model that relates the effect of genetic variation on traits to the effect of genetic variation on gene expression. It also provides unbiased estimates, detecting and correcting for propensity and reverse causation bias in causal effect estimates (36). The weighted median method weights the causal effects of different genetic variants on a trait and then takes the weighted median as the final causal effect estimate. This method is robust and can reduce bias due to deviations in the estimates of certain genetic variants. However, the criterion for using the weighted median method is that at least 50% of the SNPs must satisfy the prerequisite of valid IVs (37). A significance threshold of *p* < 0.05 was set, and the causal association results were expressed as odds ratios (ORs) and 95% confidence intervals (95% CIs).

### 2.4 Reverse MR analysis

Reverse MR analysis was performed to assess whether endometriosis affects GERD, and screening instrumental variables, Mendelian randomization analysis, and sensitivity analysis were performed sequentially. Instrumental variables were selected as described in Section “2.2 Selection of instrumental variables,” and statistical methods were selected as described in Section “2.3 Statistical methods.”

## 3 Results

### 3.1 Results of MR analysis using IVs based on genome-wide significance screening

MR results were based on instrumental variables screened at the genome-wide significance threshold (*p* < 5×10^−8^), and a total of 44 SNPs associated with confounding factors (body mass index, height, depression and anxiety, menarche, reproductive history, back pain, and the influence of economic factors) were excluded. The causal effect of GERD on endometriosis and on endometriosis occurring in different locations was assessed based on 33 instrumental variables after removing the palindromic SNPs, outcome-associated SNPs (*p* < 0.05), and SNPs that were not present in the outcome GWAS pooled data. Detailed information on the confounding SNPs associated with the results is provided in Supplementary Table 1, and detailed information on the instrumental variables for MR and the results of the analyses are provided in Supplementary Table 2.

In all endometriosis databases, the f-statistics of all IVs were greater than 10, ranging from 29.75∼45.55, which excluded the interference of weak instrumental variables on the results. In addition, the results of MR analysis for IVs screened based on genome-wide significance thresholds are shown in Table 3. The MR results indicated that there was no significant causal relationship between GERD and the occurrence of endometriosis (UK Bank: OR ≈ 0, 95% CI 1.0007–1.0044, *P* = 0.006; FinnGen: OR = 1.29, 95% CI 0.99–1.67, *P* = 0.06). In addition, MR results, occurring in the subdatabases of fallopian tubes, pelvic peritoneum, ovaries, rectovaginal septum with vagina and intestines, yielded the same conclusions as described above, and the results of MR analysis are shown in Table 3.

**TABLE 3:** MR analysis results.

Exposure	Outcome	*n* SNP	Method	OR (95% CI)	*P*-value
GERD	ENDOMETRIOSIS (UK Bank database)	33	IVW	1.001–1.004	0.006
		33	MR-Egger	0.983–1.018	0.96
		33	Weighted median	0.999–1.004	0.20
GERD	ENDOMETRIOSIS (FinnGen database)	31	IVW	0.992–1.666	0.06
		31	MR-Egger	0.202–29.965	0.49
		31	Weighted median	0.820–1.555	0.46
GERD	ENDOMETRIOSIS_FALLOPIAN_TUBE	31	IVW	0.155–4.328	0.81
		31	MR-Egger	0.000–7.18e+08	0.58
		31	Weighted median	0.098–1.01e+01	1.00
GERD	ENDOMETRIOSIS_UTERUS	31	IVW	0.999–2.166	0.05
		31	MR-Egger	0.009–13.542	0.57
		31	Weighted median	0.926–2.638	0.09
GERD	ENDOMETRIOSIS_PELVICPERITONEUM	31	IVW	0.764–1.753	0.49
		31	MR-Egger	0.072–211.151	0.51
		31	Weighted median	0.579–1.630	0.91
GERD	ENDOMETRIOSIS_OVARY	31	IVW	0.850–1.895	0.24
		31	MR-Egger	0.200–412.233	0.27
		31	Weighted median	0.695–1.787	0.65
GERD	ENDOMETRIOSIS_RECTPVAGSEPT_VAGINA	31	IVW	0.630–1.757	0.85
		31	MR-Egger	0.157–2,533.167	0.24
		31	Weighted median	0.401–1.602	0.53
GERD	ENDOMETRIOSIS_INTESTINE	31	IVW	0.263–3.886	0.99
		31	MR-Egger	8.07e–06–1.07e+06	0.87
		31	Weighted median	0.135–4.779	0.81

**FIGURE 4:**
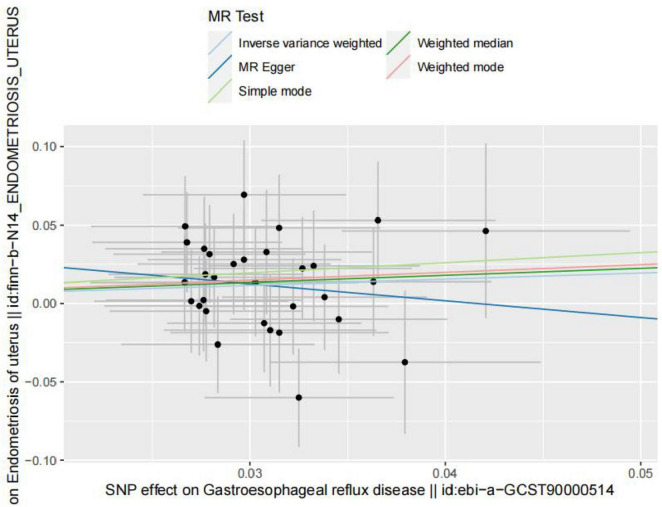
Scatterplot of genetic correlations between exposure (endometriosis occurring at the uterus) and outcome (gastroesophageal reflux disease) based on IVs screened at genome-wide significance thresholds.

However, in the subdatabase of endometriosis confined to the uterine corpus, MR results demonstrated a causal relationship between GERD and the development of endometriosis. Specifically, MR results in IVW indicated that GERD significantly increased the risk of endometriosis (OR = 1.47, 95% CI 1.00–2.17, *P* = 0.05) (Table 3). In addition, two other MR methods yielded similar causal estimates, including MR-Egger and weighted median (Table 3 and Figure 4). Sensitivity analyses were conducted to assess the robustness of the MR results. The MR Steiger test indicated that the inferred causal direction between exposure (GERD) and outcome (endometriosis) was in the “right direction” (*p* < 0.05). The Cochran’s Q test indicated that there was no heterogeneity between IVs (*p* > 0.05) (Table 4). The results of the MR- Egger intercept test and the MRPRESSO global test indicated that the MR analyses were not potentially affected by any level of pleiotropy (*p* > 0.05) (Table 5). Leave-one-out sensitivity analyses confirmed the robustness of the MR results, as there were no prior SNPs that severely affected the results upon exclusion (Supplementary Figure 1).

**TABLE 4:** Heterogeneity results of Cochran’s Q test.

Exposure	Outcome	Method	Cochran’s Q test		
			Q	Q_df	Q_pval
GERD	ENDOMETRIOSIS_UTERUS	IVW	20.54	29	0.875
GERD	ENDOMETRIOSIS_UTERUS	MR-Egger	21.15	30	0.883

**TABLE 5:** Results of MR-Egger intercept test and MR-PRESSO global test for horizontal multivariate validity.

Exposure	Outcome	MR-Egger intercept test			MR-PRESSO global test	
		Intercept	SE	*P*-value	RSS obs	*P*-value
GERD	ENDOMETRIOSIS_UTERUS	0.045	0.057	0.44	22.66	0.883

### 3.2 Reverse MR results

Reverse MR analysis of the UK Bank database and endometriosis occurring at the uterus with no valid IVs after removal of the palindromic SNPs, outcome-associated SNPs (*p* < 0.05), SNPs not present in the resultant GWAS pooled data, and SNPs associated with confounders. The FinnGen database assessed the causal effect of endometriosis on GERD based on five IVs. Detailed information on the IVs for reverse MR analysis is shown in Supplementary Tables 3, 4. None of the MR methods showed a causal relationship between endometriosis and GERD (*p* > 0.05) (Table 6). The Cochran’s Q test showed that reverse MR analysis was affected by heterogeneity (*p* < 0.05) (Table 7). In addition, the MR-Egger intercept test and MR-PRESSO global test showed that the reverse MR analysis was not affected by horizontal pleiotropy (*p* > 0.05) (Table 8). Finally, leave-one-out sensitivity analysis confirmed the robustness of the reverse MR results (Supplementary Figure 2).

**TABLE 6:** Reverse MR results of causality of occurrence and endometriosis at the uterus on GERD.

Exposure	Outcome	*n* SNP	Method	OR (95% CI)	*P*-value
ENDOMETRIOSIS (FinnGen database)	GERD	5	IVW	0.965–1.077	0.49
		5	MR-Egger	0.842–1.794	0.36
		5	Weighted median	0.979–1.074	0.30

**TABLE 7:** Heterogeneity results of Cochran’s Q test in reverse MR analysis.

Exposure	Outcome	Method	Cochran’s Q test		
			Q	Q_df	Q_pval
ENDOMETRIOSIS (FinnGen database)	GERD	IVW	29.86	7	1.01e-04
		MR-Egger	29.81	6	4.28e-05

**TABLE 8:** Horizontal multivariate results of MR-Egger intercept test and MR-PRESSO global test in MR reverse analysis.

Exposure	Outcome	MR-Egger intercept test			MR-PRESSO global test	
		Intercept	SE	*P*-value	RSS obs	*P*-value
ENDOMETRIOSIS (FinnGen database)	GERD	0.002	0.02	0.923	36.84	< 0.001

## 4 Discussion

In this study, bidirectional MR analysis was performed using a variety of MR methods, and the results showed that from the entire endometriosis dataset, no significant causality with GERD was found in either forward or reverse MR analysis (even though a significant causality was shown in the UK bank database, the OR was approximately equal to 1, suggesting that the occurrence of GERD did not significantly increase the risk of developing endometriosis). These associations were robust in sensitivity analyses, with no detectable heterogeneity or pleiotropy. The above results were largely consistent in MR analysis using IVs screened based on genome-wide significance thresholds from databases in different countries, adding more confidence to the results. Our findings are consistent with the results of previous reports on this type of disease by Adewuyi et al. (19). Surprisingly, however, when we analyzed the data using the Finn database, which provides subdatasets of endometriosis occurring in different locations, and when analyzing each subdataset individually, we found that genetically predicted GERD significantly increased the risk of endometriosis occurring in the uterine corpus, while at the same time, the reverse MR analysis revealed that confinement to the body of the uterus of the endometriosis did not appear to be causally related to GERD.

Previously reported observational studies have hinted at a possible relationship between GERD and endometriosis. Seaman et al. (38) found that endometriosis may coexist with the manifestation of gastrointestinal symptoms compared to healthy controls. Smorgick et al. (39) noted that the associations were closer relative to adolescents and young women, particularly in the adult female subgroup. Similarly, a cross-sectional cohort study involving Danish women found an association between gastrointestinal symptoms in patients with endometriosis, with cause and effect unknown (40). El Moaein and Carpentier (9) performed a clinical report showing the complex impact of a previous history of endometriosis on gastroesophageal reflux disease. In addition, the severity of gastroesophageal reflux can contribute to the development of endometriosis. For example, Mysior et al. (10) and Dasari et al. (11) reported that endometriosis involving the gastrointestinal tract should be considered when identifying acute abdominal pain in women of childbearing age. Although these observational studies do not explain causality, they provide sufficient evidence for an association between GERD and endometriosis.

Endometriosis confined to the uterine corpus, also known as adenomyosis, is a diffuse or confined lesion formed by the invasion of endometrial glands and mesenchyme into the myometrium, and its pathogenesis and pathophysiology have not yet been clarified, although it has been reported to have some genetic homology with endometriosis in other sites. The association of the study population with other diseases is shown in Table 9, from which it can be seen that the overlap between patients with adenomyosis and those with intestinal endometriosis was 2.82%, and with other gastroesophageal and gastrointestinal diseases was less than 2%. There was about 7% overlap between these study participants and those identified as being in stages 1,2 of endometriosis American Society for Reproductive Medicine (ASRM) and stages 3,4 of endometriosis ASRM. Notably, the patients with adenomyosis studied had an overlap of more than 20% with benign uterine fibroids with endometriosis and endometriosis. Meanwhile, Table 10 provides the drug use in the study population, which shows that the overlap between the adenomyosis patients and the concomitant drug group was less than 2% in all cases, with the exception of the “Triptan medication for migraine,” which also had an approximate overlap of 2%. Therefore, the conclusion of our study that gastroesophageal reflux may somewhat increase the risk of developing adenomyosis was less affected by the medications used by the study subjects, and it can be ruled out that these patients were not at pathogenic risk due to the use of medications. In the present MR study, GERD can lead to an increased risk of adenomyosis without a significant causal relationship with other sites of endometriosis, which explores the possible different pathogenesis of adenomyosis and other endometriosis from another new angle and provides a new direction for further pathologic studies.

**TABLE 9:** Association of study groups with other diseases.

Disease type	Case overlap *N* (Jaccard index)
Endometriosis of intestine	162 (2.82)
Diverticular disease of intestine	850 (1.95)
Other diseases of intestines	1,898 (1.89)
Diseases of esophagus, stomach and duodenum	1,321 (1.75)
Gastro-oesophageal reflux disease	707 (1.85)
Gastrointestinal diseases	3,711 (1.41)
Other noninfective gastroenteritis and colitis	191 (1.23)
Diarrhea and gastroenteritis of presumed infectious origin	543 (1.29)
Intestinal stricture	212 (1.22)
Paralytic ileus and intestinal obstruction	186 (1.17)
Other functional intestinal disorders	457 (1.75)
Intestinal infectious diseases	770 (1.35)
Any gastric operation	3,206 (1.65)
Benign leiomyoma with endometriosis	1,985 (21.49)
Endometriosis of rectovaginal septum and vagina	369 (4.63)
Deep endometriosis	487 (5.79)
Endometriosis diagnosis and infertility diagnosis occurring together	689 (7.85)
Unspecified/other endometriosis	560 (6.47)
Endometriosis of pelvic peritoneum	888 (7.68)
Endometriosis ASRM stages 1,2	913 (7.80)
Endometriosis of ovary	825 (6.95)
Endometriosis ASRM stages 3,4	1,033 (7.50)
Endometriosis	5,319 (28.11)

*N* indicates the number of overlapping individuals between two diseases (or study endpoints). The Jaccard index is used to measure the similarity of two sets with a value between 0 and 1, where 0 means completely different and 1 means completely the same, where the index is taken as a percentage value. Only disease types with index percentile values greater than 1 and associated with intestinal endometriosis are listed in the table; other diseases with less overlap with patient use are not listed.

**TABLE 10:** Drug use in the Finnish patient group.

Drug type	Case overlap *N* (Jaccard index)
Use of eye-antiallergens (taken as indicator of allergic/atopic conjunctivitis)	533 (1.73)
Benzodiazepine use	981 (1.65)
Use of pramipexole	195 (1.50)
Care involving use of rehabilitation procedures	572 (1.56)
Use of hypnotics and sedatives	1,421 (1.69)
Medicines for HER+breast cancer	282 (1.55)
Other (not insulin) diabetes medications	845 (1.11)
ILD medications: immunosuppressive drugs	445 (1.27)
ILD medications	1,562 (1.67)
Diabetes medication	891 (1.05)
ILD medications: prednisolone, methylprednisolone, prednisone	1,463 (1.73)
Triptan medication for migraine, single purchase ok	1,148 (2.08)
Second line medication for Crohn’s disease	386 (1.26)
Codeine or tramadol medication	1,499 (1.91)
First line medication for Crohn’s disease	1,675 (1.67)
Depression medications	2,352 (1.69)
Statin medication	2,127 (1.29)
Antihypertensive medication–note that there are other indications	3,321 (1.32)
Paracetamol of NSAID medication	5,150 (1.29)

*N* indicates the number of overlapping individuals between two diseases (or study endpoints). The Jaccard index is used to measure the similarity of two sets with a value between 0 and 1, where 0 means completely different and 1 means completely the same, where the index is taken as a percentage value. Only medication types with Index percentile values > 1 are listed in the table; other medications with low overlap of use with patients are not listed.

MR research is an innovative approach to inferring causality. Compared to traditional observational studies, MR studies eliminate confounding variables and reverse causation. Compared to randomized controlled trials, MR studies are more effective, and there are no ethical restrictions on their implementation. The MR results showed that GERD significantly increased the risk of endometriosis confined to the uterus (IVW: OR = 1.47, 95% CI 1.00–2.17, *P* = 0.05) using IVs based on genome-wide significance threshold screening. In addition, the extrapolation of the weighted median approach was consistent with the results of IVW (37). Subsequently, various sensitivity tests further demonstrated the validity of the results.

Meanwhile, the reason for analyzing the slight difference with the results of Adewuyi et al. (19) is most likely related to the exclusion of confounders in the instrumental variables, and the literature on MR analysis of GERD and endometriosis was cited in the latest review of the causal relationship between different exposures and endometriosis using Mendelian randomization, which referred to the association between GERD and depression, and GERD may play a role as a mediating variable in depression and anxiety affecting the occurrence of endometriosis, implying that previous studies did not exclude the interference of depression and anxiety as a confounding factor on the research results, and can also indicate that this paper based on the results of the latest research progress to exclude the new confounding factors may get different analytical conclusions of research and practical significance, and can provide a new reference value (41). On the basis of previous studies and conclusions, we continue to dig deeper into endometriosis occurring in different locations and find that GERD may increase the risk of adenomyosis, which can also prove the results of previous studies to a certain extent.

Several hypotheses could explain the increased risk of adenomyosis caused by GERD. First, previous studies imply that other syndromes of the intestinal tract are closely associated with endometriosis conditions (15), both showing a tendency to increase the overall level of chronic inflammation (39, 42, 43). In this context, the activation of mast cells and their degranulation, followed by the release of lymphokines, tumor necrosis factor-alpha, and the presence of proinflammatory cytokines in mesenchymal tissues, promotes the persistence of a chronic inflammatory situation (42, 44–47). Considering the pathophysiologic mechanisms shared by GERD and endometriosis, the possible diagnosis of both pathologies needs to be investigated in the presence of severe pelvic pain. Second, depression and anxiety may mediate GERD-induced endometriosis (22, 29). Since GERD episodes may lead to elevated levels of central nervous system inflammation, which may trigger depression and anxiety, patients with GERD often suffer from more severe anxiety-depression (48, 49). At the same time, anxiety depression produces estrogen disorders, which exacerbate depression anxiety and make endometriosis, which is regulated by estrogen, possible (2). Therefore, it is necessary to maintain psychological support for patients with GERD to maintain estrogen stability and reduce the risk of subsequent endometriosis.

The current study has some strengths. First, this is an MR investigation assessing the causal relationship between GERD and endometriosis, which obtained not exactly the same conclusions as previous studies and did not find a significant causal relationship between GERD and endometriosis in both the positive and negative directions. Second, the MR analyses in this paper were performed using separate pooled-level data from large-scale GWAS in different countries, which improves the confidence of inferences due to the large sample sizes and different populations, and many MR methods and sensitivity analyses were used to improve the confidence of the results. Third, thanks to the Finn database, which breaks down endometriosis occurring in different locations, the present study enriches and completes the findings of previous studies and is highly likely to imply a causal association between GERD and specific sites of endometriosis occurrence. However, this study has some limitations. First, the original GWAS pooled data analyzed in this study were from a European population; therefore, the findings may not be applicable to other ethnicities. Second, due to the limitations of the GWAS pooled data, it was not possible to stratify the analysis for general factors such as age and gender. Third, it is difficult to ensure that the results are completely independent of horizontal polymorphism effects. Therefore, we performed a series of sensitivity analyses to demonstrate the reliability of the results.

## 5 Conclusion

Evidence is provided that genetically predicted GERD increases the risk of adenomyosis. Therefore, symptomatic treatment of patients with GERD should be complemented by gynecological examination to avoid and prevent the development of endometriosis.

## Data Availability

Publicly available datasets were analyzed in this study. This data can be found here: the datasets analyzed during the current study are available in the GWAS database, https://www.ebi.ac.uk/gwas.

## Supplementary material

Supplementary Figure 1 Funnel plots of the causal effect between GERD and endometriosis confined to the uterine corpus.

Supplementary Figure 2 Funnel plots of the causal effect between endometriosis confined to the uterine corpus and GERD.
