# Supplementary figures and images for: The causal role of gastroesophageal reflux disease in endometriosis: a bidirectional Mendelian randomization study
Source: Front Med (Lausanne). 2024 Oct 30;11:1440157. doi: 10.3389/fmed.2024.1440157 (PMC11558527; doi:10.3389/fmed.2024.1440157)

# MR Method

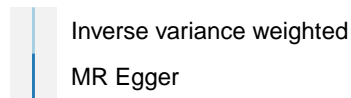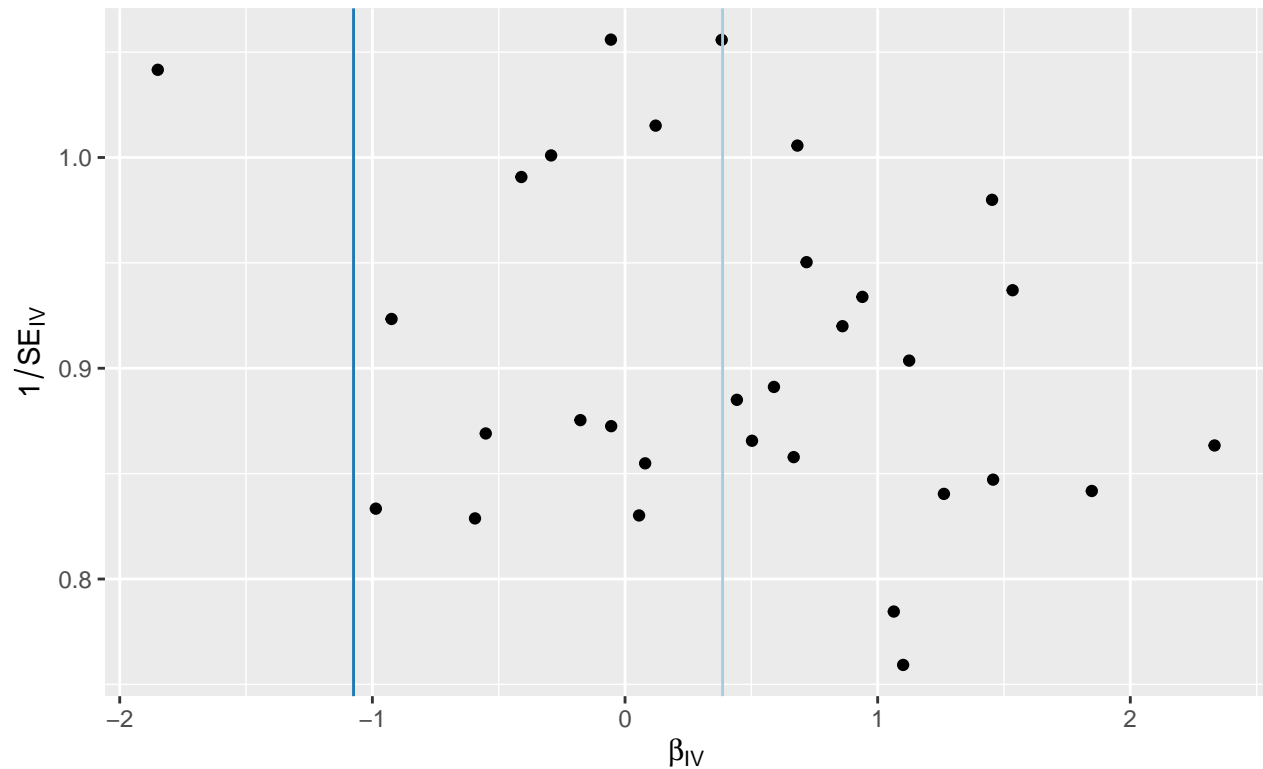

Supplement: Supplementary Figure 1 — Funnel plots of the causal effect between GERD and endometriosis confined to the uterine corpus. [file Data_Sheet_1.PDF]

# MR Method

Inverse variance weighted  
MR Egger

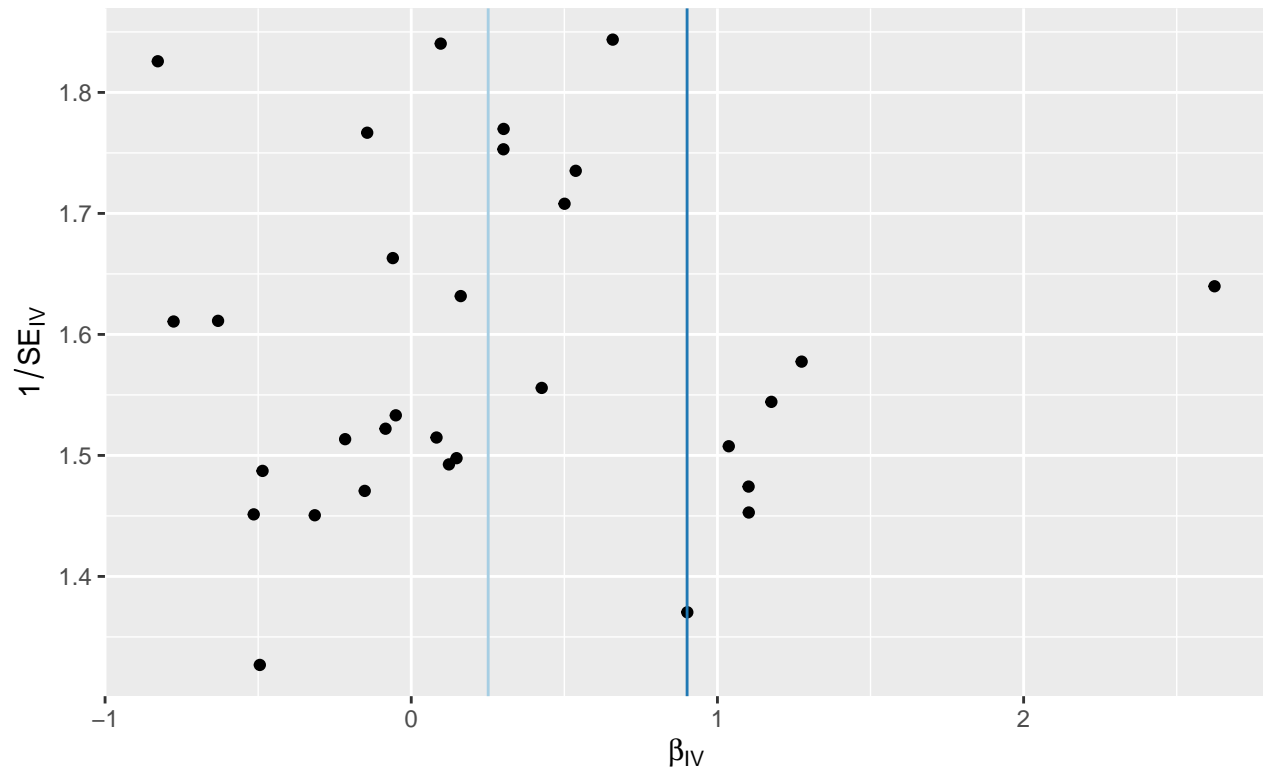

Supplement: Supplementary Figure 2 — Funnel plots of the causal effect between endometriosis confined to the uterine corpus and GERD. [file Data_Sheet_2.PDF]
